# Translational Regenerative Therapies for Chronic Spinal Cord Injury

**DOI:** 10.3390/ijms19061776

**Published:** 2018-06-15

**Authors:** Kyriakos Dalamagkas, Magdalini Tsintou, Amelia Seifalian, Alexander M. Seifalian

**Affiliations:** 1 The Institute for Rehabilitation and Research, Memorial Hermann Texas Medical Centre, Houston, TX 77030, USA; dalakir@gmail.com; 2 Centre for Nanotechnology & Regenerative Medicine, Division of Surgery and Interventional Science, University College of London (UCL), London NW3 2QG, UK; magda.tsintou@gmail.com; 3 Center for Neural Systems Investigations, Massachusetts General Hospital/HST Athinoula A., Martinos Centre for Biomedical Imaging, Harvard Medical School, Boston, MA 02129, USA; 4 Faculty of Medical Sciences, UCL Medical School, London WC1E 6BT, UK; asseifalian@yahoo.co.uk; 5 NanoRegMed Ltd. (Nanotechnology & Regenerative Medicine Commercialization Centre), The London BioScience Innovation Centre, London NW1 0NH, UK

**Keywords:** neuroregeneration, chronic spinal cord injury, central nervous system, stem cells, molecular therapies, biomaterials, nanotechnology, nanomaterial

## Abstract

Spinal cord injury is a chronic and debilitating neurological condition that is currently being managed symptomatically with no real therapeutic strategies available. Even though there is no consensus on the best time to start interventions, the chronic phase is definitely the most stable target in order to determine whether a therapy can effectively restore neurological function. The advancements of nanoscience and stem cell technology, combined with the powerful, novel neuroimaging modalities that have arisen can now accelerate the path of promising novel therapeutic strategies from bench to bedside. Several types of stem cells have reached up to clinical trials phase II, including adult neural stem cells, human spinal cord stem cells, olfactory ensheathing cells, autologous Schwann cells, umbilical cord blood-derived mononuclear cells, adult mesenchymal cells, and autologous bone-marrow-derived stem cells. There also have been combinations of different molecular therapies; these have been either alone or combined with supportive scaffolds with nanostructures to facilitate favorable cell–material interactions. The results already show promise but it will take some coordinated actions in order to develop a proper step-by-step approach to solve impactful problems with neural repair.

## 1. Introduction

Regenerative medicine is an exciting and relatively new field of medicine that is still in its infancy. Several attempts have been made for new and innovative applications in clinical practice. Chronic spinal cord injury (SCI) sets an excellent example because there are currently no interventions to restore body functions after chronic SCI, so novel regenerative interventions can be tested using several already developed, clinically relevant chronic SCI models. This can not only make a significant difference in the clinic and the patient’s overall functional outcome and quality of life, but also in the financial burden within society, given the enormous healthcare cost linked to chronic SCI [1]. Despite the severe and devastating nature of the condition, from a research point of view, chronic SCI could be perceived as a natural opportunity to utilize the potentials of regenerative therapies to overcome the boundaries that such an untreatable condition set. Due to the inherent inability of the Central Nervous System (CNS) to regenerate, chronic SCI poses a great challenge for regenerative medicine to prove its utility for real-world applications, opening the pathway for further CNS applications.

SCI is a devastating disease that results in paralysis, either immediately following the injury or in a very short period of time, depending on the cause, e.g., neurotrauma, inflammatory disease, etc. The current state-of-the-art intervention for SCI is neurorehabilitation. Surgery might initially be needed in order to stabilize the bone structure. After that the patients are transferred to a rehabilitation unit in order to learn how to take care of their basic bodily functions, e.g., bowel and bladder management, as well as in order to develop important skills that will help them reintegrate into society. Although rehabilitation is the only strategy used to manage SCI for the time being, its efficacy and reproducibility depend on many factors. For example, the individual’s personality and his perceptions may hinder his adaptation to the changes. In addition to that, the type of impairment plays a significant role too, for example, high versus low SCI lesions. Finally, another important factor to consider, which influences rehabilitation, is the type of environment/community where the person is to be reintegrated, e.g., developed versus developing countries, present or absent laws for people with disabilities in society and the needed infrastructure for an accessible environment in the community.

**Figure 1:**
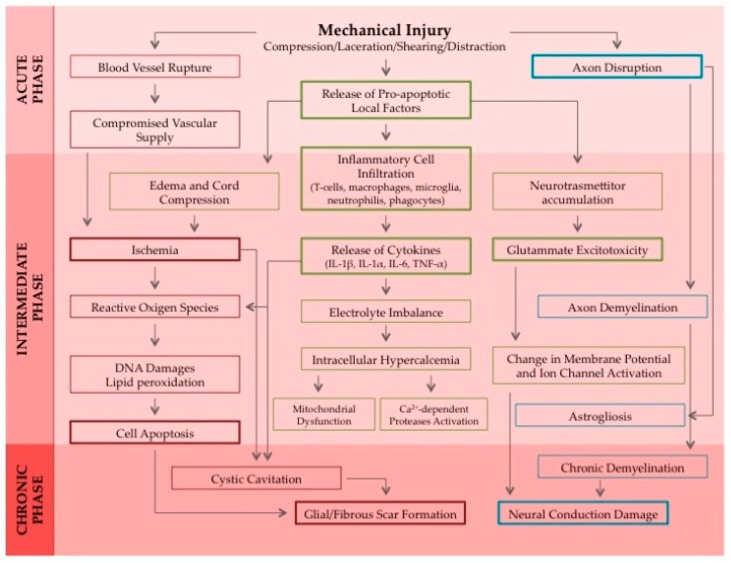
This is a schematic representation of the cascade of events that are included in the pathophysiological response to a spinal cord injury induced by a mechanical trauma. All the phases from the acute to the sub-acute and chronic SCI are being depicted until cavitation occurs in the lesion site and the glial scar forms. Abbreviations used: IL-1α: Interleukin 1α; IL-1β: Interleukin 1β; IL-6: Interleukin 6; TNF-α: Tumor Necrosis Factor α. Figure reprinted with permission from “Nanofiber Scaffolds as Drug Delivery Systems to Bridge Spinal Cord Injury” by Faccendini et al., *Pharmaceuticals* 2017, 10, 63, licensed under a Creative Commons Attribution license [4].

SCI can be divided into three phases: acute, subacute, and chronic (Figure 1). The most plausible and within reach target for regenerative medicine for proof of concept of translational applications seems to be the chronic phase, when inflammation has subsided and any kind of neural plasticity and spontaneous regeneration has already failed, making the interpretation of any results much clearer. Many scientists argue that regeneration is a major challenge after the lesion is well established in the chronic phase and this is why most of the studies focus on acute SCI, avoiding the formation of a glial scar, which would make any interventions harder. Nevertheless, it has been demonstrated before that bridging axonal regeneration in the adult CNS after a chronic SCI lesion is achievable as long as both the intrinsic growth state of the neuron and the nonpermissive established injury environment get modified [2,3].

Currently there are several clinical trials worldwide that attempt to deliver feasibility/proof of concept for regenerative therapies. Two main approaches are currently being used and they are discussed in the “Cell therapies” and “Molecular therapies” sections below in order to bring the most promising translational research for chronic SCI to the attention of the scientific community. This is crucial, given the overwhelming number of publications, reported over 11,000 within the past five years on such a promising field, leading to the inability of research work to focus on promising therapies that are closer to clinical translation. The purpose of this article is not to include an extensive list of therapeutic strategies for chronic SCI, but to focus on the ones that are the most promising for future applications in the clinic. The key aspect affecting the success of such therapeutic strategies is the use and proper choice of biomaterials for the development of 3D scaffolds to support nerve growth within the cavity lesion, as well as providing trophic factors, biomolecules, and/or cells as delivery systems. Due to their importance, we have also included a last section with selected biomaterials that we think will be excellent candidates for future clinical applications, given their promising preclinical results.

## 2. Cell Therapies

Cell-based translational therapies have attempted using types of stem cells alone or in combination with growth factors or other molecules in order to induce nerve–axon sprouting or to neutralize the growth inhibitor factors. Stem cells have also been used in conjunction with biocompatible scaffolds, which encapsulate and gradually release the cells, guiding and tuning the process of nerve growth and repair. Several clinical trials have arisen that target chronic SCI.

### 2.1. The “Pathway Study” of Stemcell Inc. (Phase I/II Clinical Trials)

The “Pathway study” [5,6] used adult neural stem cells (NSCs) derived from fetal tissue for transplantation to chronic SCI, recruiting patients with cervical SCI lesions. Unfortunately, the study had to stop due to results that were deemed too moderate for Stemcell Inc., given the funding that the study needed for its completion. This was despite the improvement noted, especially in hand function, in a few of the recruited patients after the transplantation.

Based on the company’s reports, all state and federal guidelines were followed when obtaining the human fetal brains from Advance Bioscience Resources. The company used HuCNS-SC product in a form of “neurospheres” for transplantation. This product is comprised of a highly purified population of human neural stem cells that are grown in a suspension as clusters of cells, hence why it is called “neurospheres”. The rationale for using those cells is that they can maintain their ability to self-renew and differentiate into the three major cell types of the CNS (i.e., neurons, astrocytes, and oligodendrocytes) after being cultured and expanded for a number of generations. Even though the use of the HuCNS-SC product was found to be safe and encouraging patterns of motor and sensory improvements were noted at the 6-month mark of the study, the improvement declines over time. Even though there was still improvement compared to the baseline, the results were not considered to be adequate enough to justify the cost of the study.

### 2.2. Phase I Clinical Trial of NeuralStem Inc.

NeuralStem Inc. [7] has enrolled four American Spinal Injury Association (ASIA) Impairment Scale (AIS)-A thoracic chronic SCI subjects (1–2 years post-injury at the time of stem cell treatment) for the ongoing clinical trial phase I that they are conducting for chronic SCI. The company uses human spinal cord stem cells (NSI-566), stemming from a single 8-week-old fetus. The cells are expanded serially by epigenetic means only. NSI-566 is a novel human neural stem cell line that possesses robust growth properties and neurogenic potential. In preclinical models, when the cells were grafted into a rat spinal cord, the cells differentiated extensively into neurons and glia, secreted neurotrophic factors and formed synapses with the host neural cells, but not with muscles [8,9].

Even though there are no published data available yet, the last surgery was completed in July of 2015, so the company has already conducted a 6-month post-observation analysis of the results. The company claims that the treatment was well tolerated with no serious adverse reactions. The enrolled subjects are currently being monitored for long-term follow-up evaluations. The Food and Drug Administration (FDA) has approved the protocol amendment to treat an additional cohort of four cervical SCI patients. In April 2018, Neuralstem Inc. announced the completion of the first surgery in the cervical cohort of the Phase I clinical trial in patients with chronic SCI. At the same time, NeuralStem Inc. has already proceeded with a phase I/II clinical trial to treat motor deficits in stroke patients and to establish a treatment for Amyotrophic Lateral Sclerosis (ALS). Thus, during the next decade, there is probably a lot more to be explored in terms of clinical applications of fetal stem cells.

Recently, Rosenzweig et al. [10] published a very promising research paper about the restorative effects of NeuralStem Inc. donated cells in nonhuman primate spinal cord models. In particular, they grafted spinal cord-derived neural progenitor cells (NPCs) into sites of cervical C7 hemisection spinal cord lesions 2 weeks after the hemisections surgery took place. During a 9-month analysis, forelimb function improvement was noted several months after the grafting took place, while monkey axons were found to regenerate and form synapses, suggesting translatability of the NPCs graft therapy to humans.

### 2.3. The Chronic SCI Stem Cell Study of InVivo Therapeutics

The neurospinal scaffold made by InVivo therapeutics company in USA is composed of FDA approved poly(lactic-co-glycolic acid) (PLGA) covalently conjugated to poly(l-lysine) to facilitate favorable cell–material interactions. InVivo Therapeutics utilizes injectable combinations of biomaterials and NSCs, delivered using minimally invasive surgical instrumentation and techniques to create trails across the chronic injury site.

InVivo Therapeutics has already announced several promising results on the progress of the acute SCI study, called INSPIRE [11], which has already reached to a phase III clinical trial. The company recently reported that seven of 16 (43.8%) evaluable patients in the INSPIRE study experienced an improvement in the AIS grade from baseline at six months compared to the Objective Performance Criterion (study success definition) of 25% of patients. Of these seven patients, three of five individuals who had converted from AIS A SCI (complete) to AIS B SCI (sensory incomplete) in the first six-month period of follow-up subsequently further improved to AIS C SCI (motor incomplete) within 12 to 24 months, including a recent patient who converted from AIS B to AIS C at the 12-month exam in January 2018.

Unfortunately, three deaths were witnessed during the INSPIRE study that were considered to be unrelated to the Neuro-Spinal Scaffold used and the implantation technique. Nevertheless, the company has elected, based in part on discussions with the company’s independent Data Safety Monitoring Board, to implement a temporary halt to enrolment as it engages with the FDA to determine whether any changes to patient enrolment criteria related to patients who may have a higher mortality risk or other study modifications are deemed necessary.

As a result of the temporary enrolment halt, the company anticipated completing INSPIRE enrolment in the first half of 2018 and submitting a Humanitarian Device Exemption (HDE) application in the second half of 2018. As per a recent press release of the company in March 2018, the company has received supplemental Investigational Device Exemption (IDE) approval from the US FDA for a second pivotal clinical study of the company’s Neuro-Spinal Scaffold in patients with acute SCI.

In the meantime, InVivo therapeutics took the decision to focus only on the INSPIRE study for the time being, so they announced the temporary suspension of the chronic SCI stem cell study.

### 2.4. “Walk Again Project”

Raisman and co-workers in London, who pioneered the “Walk Again Project” [12]. Unfortunately, he passed away but his work is being continued by Tabakow and co-workers in Poland, the leading neurosurgeon involved in Raisman’s project. The project focuses on the use of Olfactory Ensheathing Cells (OECs) in order to accomplish functional improvement after SCI. Even though there are many unidentified mechanisms involved, OECs have been used for years in clinical trials for CNS repair and one of their functions is thought to be helping the local propriospinal interneurons to create new circuits for bypassing the lesion. The first clinical trials of OECs have already taken place in China, Australia, and Spain in 2003, 2005, and 2006, respectively [13,14,15]. Ever since, significant progress has been accomplished and the safety of OECs transplantation in humans has been established through several phase I clinical trials [16]. Nevertheless, the need for robust, well-designed phase II clinical trials is still unmet in order to measure the efficacy of that technique. Through such future clinical trials, the technique can be optimized in order to accomplish the optimal harvesting methodology and maximize the viability of the cells after transplantation [17].

**Figure 2:**
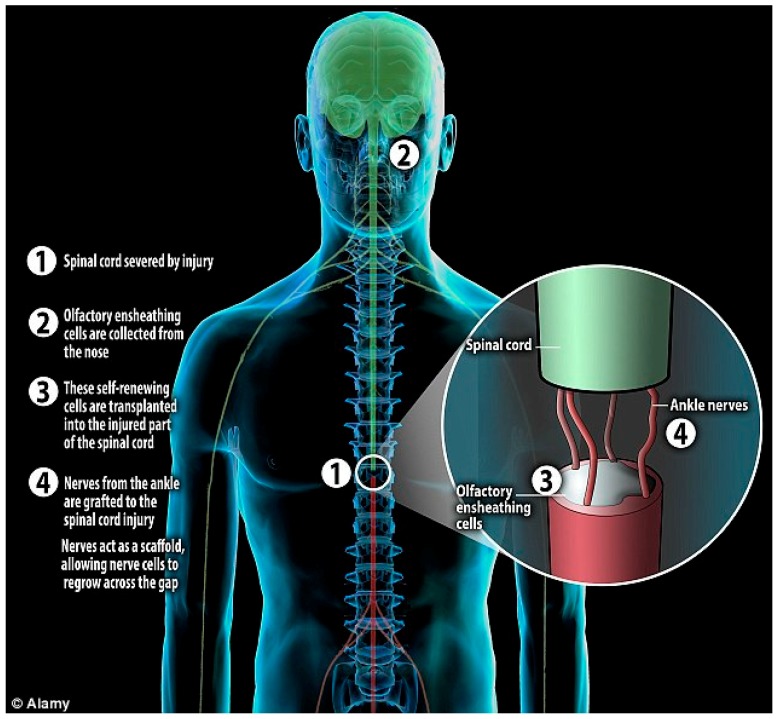
Schematic diagram shows steps of the treatment of Spinal Cord Injury (SCI) in a male patient who was paralyzed due to knife injury in 2010. He was treated with Olfactory Ensheathing Cells (OECs), a type of cell which is produced at the base of brain and through which human beings get their sense of smell. The surgeon extracted OECs from the nasal cavity and cultured those in the lab. Then nerve grafts were extracted from the ankle of the patient to support the regeneration of severed spinal cord nerve fibers to fill the spinal cavity. Both nerve grafts and stem cells were injected into the spinal cord injured site of the patient. This figure is also available online: http://www.dailymail.co.uk/sciencetech/article-2800988/world-man-spinal-cord-severed-walks-paralysed-fireman-recovers-thanks-uk-research.html.

Despite all the current limitations regarding the OECs transplantation technique, Geoffrey Raisman’s team in UCL reported very promising results, from an injured patient, in 2014 (Figure 2). As per the report, the recipient of the transplanted OECs demonstrated significant functional recovery below the level of SCI, favoring the use of OECs as an efficient treatment of SCI [12]. It is remarkable that the patient went from complete paraplegia to incomplete (ASIA A to ASIA C) and has regained considerable functions. Nevertheless, expectations from this study have to be tempered since we are now talking about a single patient. Chronic SCI patients are still (since March 2016) being recruited for a new clinical trial taking place in Poland (Tabakow and colleagues). As far as we know, the clinical trial follows the same protocol as the one that was applied to the first patient, i.e., extraction of olfactory cells from the olfactory bulb in the patient’s brain, transplantation into the spinal cord and a peripheral nerve graft. Only patients with a transected/severed spinal cord can apply for the trial and not patients with contused spinal cord.

### 2.5. “Miami Project” Phase I Clinical Trial

Schwan cells (SCs) are supporting cells surrounding the peripheral nerves. In the peripheral nervous system (PNS) they are thought to provide guidance to the axons for regeneration to take place [18]. The idea of using SCs after chronic SCI stems from the observation that SCs have been found around the lesion site after SCI [19,20], demonstrating beneficial effects. Nevertheless, it has been shown that apoptosis is a big obstacle, given the hostile CNS environment that does not favor the survival of those cells [21,22]. Despite the challenges, SCs are being used, for years now, in clinical trials targeting CNS lesions. Their safety has already been demonstrated in two already completed clinical trials involving SCs transplantation in human spinal cords. Currently, two more clinical trials phase I are in progress in Miami, Florida, USA, both studying sub-acute and chronic SCI subjects, in order to establish the safety of SCs before proceeding.

The “Miami Project” is a Phase I clinical trial for chronic SCI patients, it is currently recruiting patients and is expected to be completed by January 2019. Trial enrolment will target 2 cohorts. The study uses autologous Schwann cells harvested from the sural nerve of the participant, those cells are being transplanted into the epicenter of the participant’s SCI. The first cohort is announced to be thoracic (T) level 2–12 AIS grade A, B, or C (*n* = up to 4) and the second one to be cervical (C) level 5 through T1 AIS A, B, or C (*n* = up to 6).

### 2.6. Umbilical Cord Blood & Lithium ChinaSCINet Phase II Clinical Trial

In the fall of 2014, Wise Young, from Rutgers University and SCINetChina (available online: http://www.chinascinet.org), presented some preliminary information from the Umbilical Cord Blood & Lithium Phase II clinical trial that had taken place in China. In this trial [23], umbilical cord blood mononuclear cells (UCB-MNC) and lithium are used as a combinatorial therapy. The rationale for using lithium is that, apart from the low cost and availability in the clinic, it is known to stimulate UCB-MNC cells to secrete Nerve Growth Factor (NGF), Neurotrophin-3 (NT-3), and Glial cell line-derived Neurotrophic Factor (GDNF). In terms of the selection of UCB-MNCs, the aim is to improve recovery after chronic SCI. Several mechanisms have been proposed for the UCB-MNCs to improve recovery after CNS injury, involving secretion of anti-inflammatory cytokines [24,25,26], growth factors release [27,28,29], matrix metalloproteinase upregulation [25], tissue plasminogen activator downregulation [26], apoptosis prevention [24], mediation in myelination process [30,31], decreased gliosis [32], and increased angiogenesis [33].

Several groups have been known to attempt UCB-MNCs transplantation in patients with SCI with favorable outcomes. In the ChinaSCINet Phase I and II clinical trials [23], the patients were treated in Hong Kong (HK) and Kunming (KM) to assess the safety and efficacy of transplanting escalating doses of human leukocyte antigen (HLA)-matched UCB-MNCs into the spinal cords of people with chronic (1–19 years after) complete SCI. Wise Young explained in his presentation on the preliminary findings of the clinical trials that although none of the chronic ASIA A participants had improved motor scores, 15 out of the 20 patients were able to take steps with the aid of a walker whilst in rehabilitation.

Even though the motor scores of the chronic ASIA A patients did not improve, “functional recovery” was noted, which has raised some concerns. The main limitation is the absence of appropriate controls to assess the real effect of the UCB-MNCs transplantation. The fact that the intensive physiotherapy program was followed in combination with the stem cell transplant made it difficult to assess the real source of the improvements noted and would require further assessment. There is a possibility for the conduction of a phase IIb similar clinical trial in United States, aiming at proving the efficacy of the treatment. The structure of that new study is meant to be as follows: three groups of nine ASIA A, C5-T10 patients. The first group will get UCB-MNCs injections plus six weeks of oral lithium plus intensive rehabilitation. The second group will get UCB-MNCs plus intensive rehabilitation. Group three will get intensive rehabilitation only. This new study’s structure would certainly overcome the limitations of the previous study conducted in HK and KM.

### 2.7. The Puerta de Hierro Phase I/II Clinical Trial

In the Puerta de Hierro Phase I/II clinical trials [34], autologous bone marrow adult mesenchymal stem cells (MSCs) were used for the studies, establishing the safety of the technique. MSCs have already been correlated with beneficial outcomes when being transplanted in CNS lesions in small and big preclinical animal models, paving the way towards clinical translation. Even though MSCs were traditionally known to be able to selfrenew and differentiate into cells of mesodermal origin, they have also been found to differentiate into tissue of nonmesoderm origin (i.e., nerve tissue) and they have the potential to modulate the inflammatory response [35,36]. In that trial, the MSCs were administered by intrathecal injection (subarachnoid and intramedullary). Improvement was noted even in the patients with the longest chronicity, while the team studied both complete and incomplete chronic SCI.

In the complete chronic SCI study, the recovery noted was considered to be a result of cytokine release by the transplanted MSCs, activating preserved but nonfunctional circuits, rather than inducing nerve regeneration. This is because the recovery of infralesional sensitivity and vegetative functions (e.g., bladder, bowel, and sexual functions) occurred soon after surgery. In addition, a dose-dependent beneficial effect of the MSCs transplantation was suggested because the improvement noted in the scaled used (e.g., ASIA, International Association of Neurorestoratology Spinal Cord Injury Functional Rating Scale (IANR-SCIFRS) and neurogenic bowel dysfunction (NBD)) was more significant for higher numbers of transplanted cells.

In the incomplete chronic SCI study, the repeated subarachnoid administrations of autologous MSCs supported in autologous plasma at months 1, 4, 7, and 10 of the study improved the patients’ quality of life. Nevertheless, objective neuroimaging findings that would suggest morphological changes in the lesion site after the repeated subarachnoid administration of MSCs were absent. Therefore, the improvements were considered to be a result of the release of neurotrophic factors. It is in fact thought that the potential of MSCs transplantation for CNS regeneration relies on the ability of the MSCs to modulate the environment through their secretome. Classic growth factors and cytokines packed and secreted by the MSCs [37] are now thought to play a significant role for SCI repair, possibly by decreasing the levels of proinflammatory cytokines like Interleukine-2 (IL-2), Interleukine-6 (IL-6), and Tumor Necrosis Factor α (TNFα), among other mechanisms. This is demonstrated in a recent paper of Cizkova et al., where the molecular cocktail found in the MSCs after the MSCs transplantation in the rat SCI model was thought to be responsible for the observed motor function recovery, the attenuated inflammatory response and for the spared spinal cord tissue [38].

The Puerta de Hierro clinical trial phase II has been completed but an announcement on future trials is still pending.

### 2.8. “Neurocell” Pre-Clinical Study of Neuroplast (Phase I Clinical Trial in Preparation)

A preclinical study of Neuroplast, a company based in the Netherlands, showed that NEUROCELL (Neuroplast proprietary cells that are autologous bone-marrow-derived stem cells) significantly improved both locomotor functions and survival in those spinal cord-lesioned rats as compared to rats treated with a placebo. It is thought that autologous bone-marrow-derived stem cells can lead to functional improvement after CNS injuries by contributing towards the neuroplasticity and/or by exerting a paracrine effect. Neuroplast is currently preparing a Phase I clinical trial for chronic SCI patients. The trial will involve the transplantation of Neurocells and is expected to take place in Europe. The Neurocells are meant to have a positive effect, both in terms of neuroprotection and neuroplasticity, and thus contribute to a level of functional return in the case of both chronic and acute SCI. The first chronic SCI patients are expected to be recruited during 2018.

### 2.9. Less Strictly Regulated Clinical Trials

It should be noted that further trials in SCI patients are being conducted all over the world, but they are not performed under strict regulatory environments so caution is advised when assessing the results of such studies until further work is done in order to confirm findings in a better regulated setting [39,40,41,42,43,44,45].

## 3. Molecular Therapies

A summary of the various molecular therapies in the nanoscale projects is included in Table 1.

### 3.1. Nogo Trap of ReNetX Bio (Formerly Known as Axerion Therapeutics)

Nogo Trap is a decoy receptor developed by the ReNetX Bio company. The decoy receptor is meant to modify the hostile CNS environment by binding to the growth inhibitors within the CNS. This allows new nerve fiber growth, targeting restoration across all facets of growth: axonal regeneration (long distance), axonal sprouting (medium distance), and synaptic plasticity. The main difference to the widely-known “Anti-NoGo” technology, as per the ReNetX representatives, is that the Nogo Trap is able to bind and neutralize three types of inhibitors and is not limited to the NoGo-inhibitor alone.

Nogo Trap has demonstrated improved neurologic function following CNS damage in several animal models. Based on these promising results, the company thought that Nogo Trap should be evaluated in chronic SCI patients. ReNetX Bio is planning a phase Ib–IIa clinical trial in order to test the safety and efficacy of the treatment for patients with chronic cervical incomplete SCI.

### 3.2. CHASE-IT Preclinical Initiative of the International Spinal Research Trust (ISRT)

Chondroitinase, or Ch’ase [49], is a bacterial enzyme that has attracted the attention of neuroscientists because of its ability to degrade the glial scar tissue that develops in chronic SCI. Apart from that, it has also repeatedly been proven to promote growth and to improve recovery in preclinical animal experiments. Therefore, Ch’ase is able to modify the scar tissue that develops after SCI and promote rewiring of the nervous system. This was only made possible by the molecular re-engineering of Ch’ase, developed by Muir and colleagues [50] at the University of Cambridge, who created a version of Ch’ase that could be expressed by human cells.

Gene therapy using a modified Chondroitinase ABC (ChABC) gene compatible with expression and secretion by mammalian host cells confers sustained and long-term delivery of ChABC to the injured spinal cord. It has been shown to be effective in rats to promote functional recovery in both thoracic and cervical contusion injury paradigms [51].

Several milestones have been reached since the CHASE-IT Initiative started in 2014. In particular: (1) The gene for Ch’ase can now be expressed in an active form in human cells; (2) expression of Ch’ase in the spinal cord can now be controlled, switching it on and off using an inducible switch responsive to the antibiotic doxycycline; and (3) treatment gives rise to improved walking and unprecedented upper limb function in clinically-relevant SCI models.

## 4. Selected Biomaterials That Hold Promise for Future Clinical Trials on Chronic Sci

The application of biomaterials in SCI is divided into two strategies. The first strategy involves the application of biomaterials as a scaffold for the neuronal cells or the encapsulation of certain cells for delivery. The second strategy is using biomaterials that mimic the soft tissue mechanical properties and the high conductivity required for electrical transmission in the native spinal cord for nerve tissue regeneration.

The use of biomaterials is of great importance for CNS regeneration and repair. It was soon observed that the use of potent stem cells alone could be dangerous, given the possibility of tumor formation. It has been demonstrated that the use of certain injectable hydrogels loaded with pluripotent stem cells can promote cell survival, integration, and differentiation, thereby reducing the risk of tumorigenicity that has been linked with the use of such cell lines [52,53].

The careful choice of the appropriate biomaterial for that specific application could not only guide the process with different topographical cues, but it could also provide the necessary structural support to build a temporary bridge within the cavity that is formed in chronic SCI lesions until nerve sprouting occurs.

The use of biomaterials for CNS regeneration purposes can also use nanotechnology in order to encapsulate the cells within nanoparticle-based hydrogels and develop a sustained release system that would allow a prolonged, tuned effect to accomplish the desired outcome. Trophic factors (e.g., growth factors) can also be incorporated in a scaffold made from the appropriate biomaterial in order to support the transplanted cells to live longer or even trigger endogenous regeneration through stem cell niches.

Several biomaterials have been used for supportive scaffold formation in order to accomplish CNS regeneration after chronic SCI lesions, but so far, only limited biomaterials have made it towards clinical trials as mentioned above (e.g., PLGA). There is an overwhelming amount of different combinations of biomaterials used in chronic SCI in terms of basic science experimentation, but this would be beyond the focus of the current review. Below, we will only mention a couple of selected biomaterials that we consider to be very promising in terms of future clinical applications.

Graphene Oxide (GO) 3D nano-structured scaffolds, considered “wonder biomaterials” with extraordinary potentials in the next few years based on preclinical results:

Studies have shown graphene to have great potential as a bioscaffold at the site of the lesion in chronic SCI allowing for neuronal regeneration [4,54,55,56,57,58,59,60]. GO nanocomposite is considered to be a favorable material for use in treatment because of its unique electro-physico-chemical properties and it is conductivity. GO has the ability to stimulate neuronal differentiation and axonal alignment at sites of SCI by providing a space for the growth, attachment, and survival of neural tissue at the lesion. Toxicity and biocompatibility of reduced graphene oxide is a debatable obstacle facing use of the material, with intravenous studies in mice showing dose-dependent toxicity and pathological damage present at lower doses [61,62]. Other routes of administration, however, such as oral [63], intravitreal [64], intraperitoneal [63], and subcutaneous [65], have proven the material to be nontoxic. The conductive properties of GO make it a viable product to use in the healing of SCI. The benefit of using GO is that the inflammatory response seen with other biomaterials is reduced and not as damaging at the site of the SCI.

GO combined with hydrogel has been used to fill the hemispinal cord transection lesion that was made in twenty rats [66]. After three months, histologic evaluation of the lesion in the spinal cords of the rats showed graphene nanoscaffolds adhering to the spinal cord tissue and an ingrowth of connective tissue elements, blood vessels, neurofilaments, and Schwann cells around the area of the graphene nanoscaffolds. A control study was carried out whereby similar rats with hemispinal cord transections had a hydrogel-only matrix injected into the lesion at site of injury. Three months later, histological evaluation showed pseudocyst cavities where the hydrogel matrix had been injected and the site of the lesion devoid of any tissue or substantial regrowth of neural cells. Even though this was a study on acute SCI, this in vivo preclinical study brings promise that the graphene nanoscaffolds material has potential to be used for stimulation of axonal regeneration into the lesion.

Another aspect stressing the significance and potential of Graphene, in terms of future clinical SCI repair-related applications, is the fact that Graphene has been chosen as the key biomaterial to be used as part of the project “Neurofibers” (Biofunctionalised Electroconducting Microfibers for the Treatment of Spinal Cord Injury). The project has recently started and it was selected by the European Commission in the framework of the Horizon 2020 (H2020) program in the area of emerging technologies (FET Proactive—Boosting emerging technologies) (for more information from the European Commission’s website: https://cordis.europa.eu/project/rcn/206185_en.html).

### 4.1. Fibrin-Based Scaffolds and Hydrogels Have Shown Impressive Results in Terms of Supporting CNS Regeneration in SCI Lesions in the Right Settings

The Fibrin glue has been approved by FDA and it is used successfully in clinically repairing cranial nerves and other tissues [67,68]. The Fibrin sealant (like the commercially available TISSEEL^®^ (Baxter) product) has been used for years by neurosurgeons as a hemostatic agent and in order to control cerebrospinal fluid (CSF) leaks. This is of particular importance for spinal surgeries given the CSF leakage that can occur after the durotomy. In human patients, Fibrin has also been combined with FGF and the mixture was applied to the injured spinal segment of patients in order to prevent postoperative CSF leakage. The application of the FGF-containing Fibrin matrices resulted in significant motor and sensory improvements in the patients [69]. In terms of CNS repair and regenerative medicine, Fibrin could act as a carrier for therapeutic agents, such as neurotrophic factors and stem cells [70,71,72].

Fibrin matrices have been tested for supporting stem cells, specially embedded NSCs in fibrin matrices in order to increase the cells’ viability, when transplanted after SCI [73]. Even though the increase in NSCs viability was significant compared to the initial poor survival of the cells without the fibrin matrices, the results were even more remarkable when growth factor cocktails were added in the NSCs-containing fibrin matrices. This way the combination of NSCs with fibrin matrices and growth factors accomplished enhanced cells survival with the cells filling large lesion cavities and being differentiated into neurons and glia after spinal cord transection [2,73,74]. In a very recent paper, Rosenzweig et al. [10] used a similar methodology in nonhuman primate models, proving for one more time that testing a promising treatment in nonhuman primates is crucial for the successful translation to humans. After several modifications to the rodent grafting technique (e.g., grafting matrix modifications, CSF drainage, more extensive immunosuppression), successful engraftment was achieved, paving the path towards clinical translation of the proposed therapy. The same group of researchers had accomplished before that the longest axonal sprouting, to the best of our knowledge, using a cocktail of growth factors and fibrin matrices. Ten growth factors were embedded in a fibrin gel to support rat or human neural stem cells grafted to the completely transected spinal cord of adult rats, accomplishing axons extending at least 25 mm in each direction in all subjects [75].

Further combinatorial approaches have been attempted using Fibrin with certain growth factors and grafts to enhance the restorative results with very promising results.

The FGF/Fibrin mixture along with human Schwann-cell grafts has been engrafted to transected rat spinal cords, stimulating fiber regeneration throughout the implant [76]. This also has been coupled with an autologous peripheral intercostal nerve segment to bridge a 5 mm gap within the transected rat spinal cords [77]. Even though this is only a small part of the literature supporting the use of Fibrin for CNS repair, it is evident that this biomaterial is also a very good candidate for future clinical applications in terms of regenerative therapeutic strategies in chronic SCI.

### 4.2. Collagen/Heparin Sulfate Scaffolds Fabricated by a 3D Bioprinter

One promising, new approach is the use of a 3D bioprinter in producing collagen and heparin sulfate based bioscaffolds for the treatment of SCI [78]. The use of a 3D bioprinter, in order to produce the bioscaffold, is thought to have significantly amplified the mechanical properties of the mixture when compared to methods of production without the use of a 3D printer therefore methods using a 3D bioprinter will be discussed here. The current priorities when producing a bioscaffold for SCI is the biocompatibility of the scaffold, that it is made of a porous material in order to allow for neural regeneration and for it to have great strength. The team working on the 3D bioprinted collagen/heparin sulfate scaffolds believes it will be the answer to treating and stimulating neural regeneration in patients with SCI.

Locomotor recovery is the most important outcome to assess during preclinical and clinical studies and was seen in rats with SCI that had had the collagen/heparin sulfate bioscaffold implanted at the site of the lesion, during preclinical in vivo studies. The improved locomotor function after implantation and biodegradable and biocompatible properties of the collagen/heparin sulfate mixture gives promise for the use of such a bioscaffold in clinical practice to help improve the outcome of patients suffering with SCI.

### 4.3. Peripheral Nerve Grafts Combined with Chitosan-Laminin Scaffold

Chitosan is a suitable biomaterial for use in neuronal repair due to its biocompatible and biodegradable properties [79]. It has been considered as a suitable material for many biomedical and industrial applications, such as drug delivery, due to its nontoxicity and biodegradability. Laminin is a glycoprotein that acts as a neurite outgrowth-promoting factor and so is suitable for combination in the bioscaffold. The combination of chitosan and laminin provide a promising biomaterial for use as a scaffold in promoting axonal growth and preventing neural degeneration.

Studies have shown that the use of chitosan channels containing nerve grafts promote axonal regeneration when applied to chronic SCI lesions [80]. Preclinical studies have shown that the use of chitosan-laminin scaffolds combined with peripheral nerve grafts supported axonal regeneration and positive outcomes include motor function improvement, as well as functional sensory improvement when the bioscaffolds were implanted in chronic nerve lesions [40]. Further investigation of this biomaterial would be recommended as it proves to be a promising option in the field of treatment of chronic SCI.

## 5. Conclusions and Future Perspectives

In conclusion, it is evident that more promising therapies will come up in the future regarding chronic SCI. We anticipate that the management of chronic SCI will change during the next few decades due to the fast pace of advances in the field of nanotechnology/smart materials and regenerative medicine. A combinatorial approach using cells and/or growth factors or other molecules along with biocompatible nanostructured scaffolds, that would allow fine-tuning of the release of the incorporated factors and would guide nerve growth in the CNS environment, would most probably be the key for success in such a complex tissue.

One significant component seems to be the ability to catalyze the translation of all the promising new therapies into clinical practice. This refers to imaging technology, and more specifically, Magnetic Resonance Imaging (MRI) sequences that can help assess and objectively quantify the biological response of the CNS to the tested intervention, solving a known issue of reproducibility and quantification in the application of all the new therapies. MRI could assess the biological significance, detecting tissue-related changes, while techniques like surface electromyography could assess the functional outcomes in a more objective way, leading together to the development of the much needed objective clinical scales that would take into consideration the statistical, biological and clinical significance associated with the tested therapeutic strategy or management plan. In addition, the combination of imaging technology along with the implementation of new, clinically relevant models, like the nonhuman primate model of SCI developed for evaluating pharmacologic treatments, and could open the pathway to safer and more efficient clinical application to patients in the future [81]. Nevertheless, we do anticipate that the use of bioengineered models on-a-chip and further advancements in nanomedicine might revolutionize the field and change the translational pathway in the future, accelerating the drug approval process and the implementation of new treatments in the clinic.

From the practical standpoint, there are several obstacles that need to be tackled, like the lack of published data from companies that have done significant work on SCI regeneration and repair through clinical trials. The inclusion of controls is crucial for obtaining reliable outcomes and yet certain clinical trials either fail to implement controls in their study plan or they avoid reporting the outcomes in a timely manner, hindering the progress in the field. In addition to that, researchers mainly use less clinically relevant SCI models like hemisections/transection models. There is a significant need for inclusion of contusion SCI models that are more similar to the lesions usually managed in the clinic. Last but not least, it should be stressed that acute SCI models are mainly used for research purposes aiming to address the problem soon after the injury in the clinic and to avoid complications (e.g., formation of glial scar that would hinder neuroregeneration). The inclusion of more chronic SCI models in research might seem to be a challenging task, but it is very important for the reliable assessment of the therapeutic interventions in order to solve significant questions on CNS regeneration, ensuring the safe application of future treatments to any SCI patient.

## Figures and Tables

**Table 1:** Summary of the various molecular therapies in the nanoscale projects that show promise for the treatment of SCI. Keys: CNS: Central Nervous System; SCI: spinal cord injury; Ch’ase: Chondroitinase.

Project Name	Mechanism	Current Progress	Future Outlook	Ref.
Nogo Trap of ReNetX Bio	A decoy receptor that binds growth inhibitors, allowing for the nerve fibers to grow naturally and directly.	Nogo Trap has demonstrated improved neurologic function following CNS damage in several animal models.	Planning phase Ib–IIa clinical trials to test safety and efficacy for patients with a chronic cervical incomplete SCI.	[46]
CHASE-IT Preclinical Initiative of the International Spinal Research Trust (ISRT)	The application of the biological enzyme Ch’ase in animal models is reported to have degraded scar tissue, promoted growth and improved activity.	Ch’ase has proven to be effective in rats, delivered to both thoracic and cervical contusion injury sites. Latest animal studies took place in 2016 and proved that longer-term application of the enzyme led to more significant motor control improvement.	Promising outcome, but one should bear in mind that data is based on rodent in vivo models; will this translate to humans?	[47]
Intracellular sigma peptide (ISP), Ch’ase and combinations preclinical projects	Using the biological enzyme Ch’ase in combinations with intracellular sigma peptide in order to restore breathing after long chronic C2 hemisection injury.	These projects are at a single center led by Jerry Silver. Currently these applications are at a pre-clinical stage.	Development of the product and preparation for clinical trials.	[48]

## References

[1] National Spinal Cord Injury Statistical Center (2017). Spinal Cord Injury (SCI) Facts and Figures at a Glance.

[2] Kadoya K., Tsukada S., Lu P., Coppola G., Geschwind D., Filbin M., Blesch A., Tuszynski M.H. (2009). Combined Intrinsic and Extrinsic Neuronal Mechanisms Facilitate Bridging Axonal Regeneration One Year After Spinal Cord Injury. Neuron.

[3] Gelain F., Panseri S., Antonini S., Cunha C., Donega M., Lowery J., Taraballi F., Cerri G., Montagna M., Baldissera F. (2011). Transplantation of Nanostructured Composite Scaffolds Results in the Regeneration of Chronically Injured Spinal Cords. ACS Nano.

[4] Faccendini A., Vigani B., Rossi S., Sandri G., Bonferoni M.C., Caramella C.M., Ferrari F. (2017). Nanofiber Scaffolds as Drug Delivery Systems to Bridge Spinal Cord Injury. Pharmaceuticals.

[5] StemCells Inc. (2015). Pathway Study.

[6] StemCells Inc. (2015). Phase II Trial in Cervical Spinal Cord Injury (SCI).

[7] NeuralStem Inc. (2015). Neuralstem Reports Third Quarter 2015 Financial Results—Nov 9, 2015.

[8] Yan J., Xu L., Welsh A.M., Hatfield G., Hazel T., Johe K., Koliatsos V.E. (2007). Extensive neuronal differentiation of human neural stem cell grafts in adult rat spinal cord. PLoS Med..

[9] Xu L., Yan J., Chen D., Welsh A.M., Hazel T., Johe K., Hatfield G., Koliatsos V.E. (2006). Human neural stem cell grafts ameliorate motor neuron disease in SOD-1 transgenic rats. Transplantation.

[10] Rosenzweig E.S., Brock J.H., Lu P., Kumamaru H., Salegio E.A., Kadoya K., Weber J.L., Liang J.J., Moseanko R., Hawbecker S. (2018). Restorative effects of human neural stem cell grafts on the primate spinal cord. Nat. Med..

[11] Theodore N., Hlubek R., Danielson J., Neff K., Vaickus L., Ulich T.R., Ropper A.E. (2016). First Human Implantation of a Bioresorbable Polymer Scaffold for Acute Traumatic Spinal Cord Injury: A Clinical Pilot Study for Safety and Feasibility. Neurosurgery.

[12] Tabakow P., Raisman G., Fortuna W., Czyz M., Huber J., Li D., Szewczyk P., Okurowski S., Miedzybrodzki R., Czapiga B. (2014). Functional regeneration of supraspinal connections in a patient with transected spinal cord following transplantation of bulbar olfactory ensheathing cells with peripheral nerve bridging. Cell Transpl..

[13] Huang H., Chen L., Wang H., Xiu B., Li B., Wang R., Zhang J., Zhang F., Gu Z., Li Y. (2003). Influence of patients’ age on functional recovery after transplantation of olfactory ensheathing cells into injured spinal cord injury. Chin. Med. J..

[14] Féron F., Perry C., Cochrane J., Licina P., Nowitzke A., Urquhart S., Geraghty T., Mackay-Sim A. (2005). Autologous olfactory ensheathing cell transplantation in human spinal cord injury. Brain.

[15] Lima C., Pratas-Vital J., Escada P., Hasse-Ferreira A., Capucho C., Peduzzi J.D. (2006). Olfactory Mucosa Autografts in Human Spinal Cord Injury: A Pilot Clinical Study. J. Spinal Cord Med..

[16] Li L., Adnan H., Xu B., Wang J., Wang C., Li F., Tang K. (2015). Effects of transplantation of olfactory ensheathing cells in chronic spinal cord injury: A systematic review and meta-analysis. Eur. Spine J..

[17] Choi D., Gladwin K. (2015). Olfactory Ensheathing Cells: Part II—Source of Cells and Application to Patients. World Neurosurg..

[18] Son Y.-J., Thompson W.J. (1995). Schwann cell processes guide regeneration of peripheral axons. Neuron.

[19] Bruce J.H., Norenberg M.D., Kraydieh S., Puckett W., Marcillo A., Dietrich D. (2000). Schwannosis: Role of gliosis and proteoglycan in human spinal cord injury. J. Neurotrauma.

[20] Guest J.D., Hiester E.D., Bunge R.P. (2005). Demyelination and Schwann cell responses adjacent to injury epicenter cavities following chronic human spinal cord injury. Exp. Neurol..

[21] Bunge M.B., Wood P.M. (2012). Realizing the maximum potential of Schwann cells to promote recovery from spinal cord injury. Handb. Clin. Neurol..

[22] Wiliams R.R., Bunge M.B. (2012). Schwann cell transplantation: A repair strategy for spinal cord injury?. Prog. Brain Res..

[23] Zhu H., Poon W., Liu Y., Leung G.K.-K., Wong Y., Feng Y., Ng S.C.P., Tsang K.S., Sun D.T.F., Yeung D.K. (2016). Phase I–II Clinical Trial Assessing Safety and Efficacy of Umbilical Cord Blood Mononuclear Cell Transplant Therapy of Chronic Complete Spinal Cord Injury. Cell Transpl..

[24] Dasari V.R., Veeravalli K.K., Tsung A.J., Gondi C.S., Gujrati M., Dinh D.H., Rao J.S. (2009). Neuronal Apoptosis Is Inhibited by Cord Blood Stem Cells after Spinal Cord Injury. J. Neurotrauma.

[25] Veeravalli K.K., Dasari V.R., Tsung A.J., Dinh D.H., Gujrati M., Fassett D., Rao J.S. (2009). Human umbilical cord blood stem cells upregulate matrix metalloproteinase-2 in rats after spinal cord injury. Neurobiol. Dis..

[26] Veeravalli K.K., Dasari V.R., Tsung A.J., Dinh D.H., Gujrati M., Fassett D., Rao J.S. (2009). Stem Cells Downregulate the Elevated Levels of Tissue Plasminogen Activator in Rats After Spinal Cord Injury. Neurochem. Res..

[27] Kao C.-H., Chen S.-H., Chio C.-C., Lin M.-T. (2008). Human Umbilical Cord Blood-derived CD34+ cells may attenuate spinal cord injury by stimulating vascular endothelial and neurotrophic factors. Shock.

[28] Chua S.J., Bielecki R., Yamanaka N., Fehlings M.G., Rogers I.M., Casper R.F. (2010). The Effect of Umbilical Cord Blood Cells on Outcomes After Experimental Traumatic Spinal Cord Injury. Spine.

[29] Chung H., Chung W., Lee J.-H., Chung D.-J., Yang W.-J., Lee A.-J., Choi C.-B., Chang H.-S., Kim D.-H., Suh H.J. (2016). Expression of neurotrophic factors in injured spinal cord after transplantation of human-umbilical cord blood stem cells in rats. J. Vet. Sci..

[30] Dasari V.R., Spomar D.G., Gondi C.S., Sloffer C.A., Saving K.L., Gujrati M., Rao J.S., Dinh D.H. (2007). Axonal Remyelination by Cord Blood Stem Cells after Spinal Cord Injury. J. Neurotrauma.

[31] Cho S.-R., Yang M.S., Yim S.H., Park J.H., Lee J.E., Eom Y., Jang I.K., Kim H.E., Park J.S., Kim H.O. (2008). Neurally induced umbilical cord blood cells modestly repair injured spinal cords. NeuroReport.

[32] Ryu H.-H., Byeon Y.-E., Park S.-S., Kang B.-J., Seo M.-S., Park S.-B., Kim W.H., Kang K.-S., Kweon O.-K. (2011). Immunohistomorphometric Analysis of Transplanted Umbilical Cord Blood-Derived Mesenchymal Stem Cells and The Resulting Anti-Inflammatory Effects on Nerve Regeneration of Injured Canine Spinal Cord. Tissue Eng. Regen. Med..

[33] Ning G., Tang L., Wu Q., Li Y., Li Y., Zhang C., Feng S. (2013). Human umbilical cord blood stem cells for spinal cord injury: Early transplantation results in better local angiogenesis. Regen. Med..

[34] Vaquero J., Zurita M., Rico M.A., Bonilla C., Aguayo C., Montilla J., Bustamante S., Carballido J., Marin E., Martinez F. (2016). An approach to personalized cell therapy in chronic complete paraplegia: The Puerta de Hierro phase I/II clinical trial. Cytotherapy.

[35] Deans R.J., Moseley A.B. (2000). Mesenchymal stem cells. Exp. Hematol..

[36] Kopen G.C., Prockop D.J., Phinney D.G. (1999). Marrow stromal cells migrate throughout forebrain and cerebellum, and they differentiate into astrocytes after injection into neonatal mouse brains. Proc. Natl. Acad. Sci. USA.

[37] Hofer H.R., Tuan R.S. (2016). Secreted trophic factors of mesenchymal stem cells support neurovascular and musculoskeletal therapies. Stem Cell Res. Ther..

[38] Cizkova D., Cubinkova V., Smolek T., Murgoci A.-N., Danko J., Vdoviakova K., Humenik F., Cizek M., Quanico J., Fournier I. (2018). Localized Intrathecal Delivery of Mesenchymal Stromal Cells Conditioned Medium Improves Functional Recovery in a Rat Model of Spinal Cord Injury. Int. J. Mol. Sci..

[39] Bansal H., Verma P., Agrawal A., Leon J., Sundell I.B., Koka P.S. (2016). Autologous Bone Marrow-Derived Stem Cells in Spinal Cord Injury. J. Stem Cells.

[40] Amr S.M., Gouda A., Koptan W.T., Galal A.A., Abdel-Fattah D.S., Rashed L.A., Atta H.M., Abdel-Aziz M.T. (2014). Bridging defects in chronic spinal cord injury using peripheral nerve grafts combined with a chitosan-laminin scaffold and enhancing regeneration through them by co-transplantation with bone-marrow-derived mesenchymal stem cells: Case series of 14 patients. J. Spinal Cord Med..

[41] Frolov A.A., Bryukhovetskiy A.S. (2012). Effects of hematopoietic autologous stem cell transplantation to the chronically injured human spinal cord evaluated by motor and somatosensory evoked potentials methods. Cell Transpl..

[42] El-Kheir W.A., Gabr H., Awad M.R., Ghannam O., Barakat Y., Farghali H.A.M.A., El Maadawi Z.M., Ewes I., Sabaawy H.E. (2014). Autologous bone marrow-derived cell therapy combined with physical therapy induces functional improvement in chronic spinal cord injury patients. Cell Transpl..

[43] Wong Y.W., Tam S., So K.F., Chen J.Y.H., Cheng W.S., Luk K.D.K., Tang S.W., Young W. (2011). A three-month, open-label, single-arm trial evaluating the safety and pharmacokinetics of oral lithium in patients with chronic spinal cord injury. Spinal Cord.

[44] Cristante A.F., Barros-Filho T.E.P., Tatsui N., Mendrone A., Caldas J.G., Camargo A., Alexandre A., Teixeira W.G.J., Oliveira R.P., Marcon R.M. (2009). Stem cells in the treatment of chronic spinal cord injury: Evaluation of somatosensitive evoked potentials in 39 patients. Spinal Cord.

[45] Moviglia G.A., Fernandez Viña R., Brizuela J.A., Saslavsky J., Vrsalovic F., Varela G., Bastos F., Farina P., Etchegaray G., Barbieri M. (2006). Combined protocol of cell therapy for chronic spinal cord injury. Report on the electrical and functional recovery of two patients. Cytotherapy.

[46] ReNetX ReNetX Bio Launched to Advance Innovative Neuro-Regenerative Technology Developed at Yale University. http://globenewswire.com/news-release/2017/07/24/1056062/0/en/ReNetX-Bio-Launched-to-Advance-Innovative-Neuro-Regenerative-Technology-Developed-at-Yale-University.html.

[47] CHASE IT. https://www.spinal-research.org/chase-it.

[48] Tran A.P., Sundar S., Yu M., Lang B.T., Silver J. (2018). Modulation of receptor protein tyrosine phosphatase sigma increases chondroitin sulfate proteoglycan degradation through Cathepsin B secretion to enhance axon outgrowth. J. Neurosci..

[49] Bartus K., James N.D., Didangelos A., Bosch K.D., Verhaagen J., Yáñez-Muñoz R.J., Rogers J.H., Schneider B.L., Muir E.M., Bradbury E.J. (2014). Large-scale chondroitin sulfate proteoglycan digestion with chondroitinase gene therapy leads to reduced pathology and modulates macrophage phenotype following spinal cord contusion injury. J. Neurosci..

[50] Muir E., Raza M., Ellis C., Burnside E., Love F., Heller S., Elliot M., Daniell E., Dasgupta D., Alves N. (2017). Trafficking and processing of bacterial proteins by mammalian cells: Insights from chondroitinase ABC. PLoS ONE.

[51] James N.D., Shea J., Muir E.M., Verhaagen J., Schneider B.L., Bradbury E.J. (2015). Chondroitinase gene therapy improves upper limb function following cervical contusion injury. Exp. Neurol..

[52] Oliveira J.M., Carvalho L., Silva-Correia J., Vieira S., Majchrzak M., Lukomska B., Stanaszek L., Strymecka P., Malysz-Cymborska I., Golubczyk D. (2018). Hydrogel-based scaffolds to support intrathecal stem cell transplantation as a gateway to the spinal cord: Clinical needs, biomaterials, and imaging technologies. NPJ Regen. Med..

[53] Führmann T., Tam R.Y., Ballarin B., Coles B., Elliott Donaghue I., van der Kooy D., Nagy A., Tator C.H., Morshead C.M., Shoichet M.S. (2016). Injectable hydrogel promotes early survival of induced pluripotent stem cell-derived oligodendrocytes and attenuates longterm teratoma formation in a spinal cord injury model. Biomaterials.

[54] López-Dolado E., González-Mayorga A., Gutiérrez M.C., Serrano M.C. (2016). Immunomodulatory and angiogenic responses induced by graphene oxide scaffolds in chronic spinal hemisected rats. Biomaterials.

[55] Mattei T.A. (2014). How graphene is expected to impact neurotherapeutics in the near future. Expert Rev. Neurother..

[56] Domínguez-Bajo A., González-Mayorga A., López-Dolado E., Serrano M.C. (2017). Graphene-Derived Materials Interfacing the Spinal Cord: Outstanding in Vitro and in Vivo Findings. Front. Syst. Neurosci..

[57] Zhou K., Motamed S., Thouas G.A., Bernard C.C., Li D., Parkington H.C., Coleman H.A., Finkelstein D.I., Forsythe J.S. (2016). Graphene Functionalized Scaffolds Reduce the Inflammatory Response and Supports Endogenous Neuroblast Migration when Implanted in the Adult Brain. PLoS ONE.

[58] González-Mayorga A., López-Dolado E., Gutiérrez M.C., Collazos-Castro J.E., Ferrer M.L., del Monte F., Serrano M.C. (2017). Favorable Biological Responses of Neural Cells and Tissue Interacting with Graphene Oxide Microfibers. ACS Omega.

[59] Singh Z. Applications and Toxicity of Graphene Family Nanomaterials and Their Composites. https://www.dovepress.com/applications-and-toxicity-of-graphene-family-nanomaterials-and-their-c-peer-reviewed-fulltext-article-NSA.

[60] Kim C.-Y., Sikkema W.K.A., Hwang I.-K., Oh H., Kim U.J., Lee B.H., Tour J.M. (2016). Spinal cord fusion with PEG-GNRs (TexasPEG): Neurophysiological recovery in 24 hours in rats. Surg. Neurol. Int..

[61] Mendonça M.C.P., Soares E.S., de Jesus M.B., Ceragioli H.J., Batista Â.G., Nyúl-Tóth Á., Molnár J., Wilhelm I., Maróstica M.R., Krizbai I. (2016). PEGylation of Reduced Graphene Oxide Induces Toxicity in Cells of the Blood-Brain Barrier: An in Vitro and in Vivo Study. Mol. Pharm..

[62] Zhang X., Yin J., Peng C., Hu W., Zhu Z., Li W., Fan C., Huang Q. (2011). Distribution and biocompatibility studies of graphene oxide in mice after intravenous administration. Carbon.

[63] Yang K., Gong H., Shi X., Wan J., Zhang Y., Liu Z. (2013). In vivo biodistribution and toxicology of functionalized nano-graphene oxide in mice after oral and intraperitoneal administration. Biomaterials.

[64] Yan L., Wang Y., Xu X., Zeng C., Hou J., Lin M., Xu J., Sun F., Huang X., Dai L. (2012). Can Graphene Oxide Cause Damage to Eyesight?. Chem. Res. Toxicol..

[65] Sahu A., Il Choi W., Tae G. (2012). A stimuli-sensitive injectable graphene oxide composite hydrogel. Chem. Commun..

[66] Palejwala A.H., Fridley J.S., Mata J.A., Samuel E.L.G., Luerssen T.G., Perlaky L., Kent T.A., Tour J.M., Jea A. (2016). Biocompatibility of reduced graphene oxide nanoscaffolds following acute spinal cord injury in rats. Surg. Neurol. Int..

[67] Wieken K., Angioi-Duprez K., Lim A., Marchal L., Merle M. (2003). Nerve anastomosis with glue: Comparative histologic study of fibrin and cyanoacrylate glue. J. Reconstr. Microsurg..

[68] Brodbaker E., Bahar I., Slomovic A.R. (2008). Novel use of fibrin glue in the treatment of conjunctivochalasis. Cornea.

[69] Wu J.-C., Huang W.-C., Chen Y.-C., Tu T.-H., Tsai Y.-A., Huang S.-F., Huang H.-C., Cheng H. (2011). Acidic fibroblast growth factor for repair of human spinal cord injury: A clinical trial. J. Neurosurg. Spine.

[70] Iwakawa M., Mizoi K., Tessler A., Itoh Y. (2001). Intraspinal implants of fibrin glue containing glial cell line-derived neurotrophic factor promote dorsal root regeneration into spinal cord. Neurorehabil. Neural Repair.

[71] Cheng H., Huang S.S., Lin S.M., Lin M.J., Chu Y.C., Chih C.L., Tsai M.J., Lin H.C., Huang W.C., Tsai S.K. (2005). The neuroprotective effect of glial cell line-derived neurotrophic factor in fibrin glue against chronic focal cerebral ischemia in conscious rats. Brain Res..

[72] Petter-Puchner A.H., Froetscher W., Krametter-Froetscher R., Lorinson D., Redl H., van Griensven M. (2007). The long-term neurocompatibility of human fibrin sealant and equine collagen as biomatrices in experimental spinal cord injury. Exp. Toxicol. Pathol..

[73] Lu P., Graham L., Wang Y., Wu D., Tuszynski M. (2014). Promotion of Survival and Differentiation of Neural Stem Cells with Fibrin and Growth Factor Cocktails after Severe Spinal Cord Injury. J. Vis. Exp..

[74] Willerth S.M., Faxel T.E., Gottlieb D.I., Sakiyama-Elbert S.E. (2007). The Effects of Soluble Growth Factors on Embryonic Stem Cell Differentiation Inside of Fibrin Scaffolds. Stem Cells.

[75] Lu P., Wang Y., Graham L., McHale K., Gao M., Wu D., Brock J., Blesch A., Rosenzweig E.S., Havton L.A. (2012). Long-Distance Growth and Connectivity of Neural Stem Cells after Severe Spinal Cord Injury. Cell.

[76] Guest J.D., Hesse D., Schnell L., Schwab M.E., Bunge M.B., Bunge R.P. (1997). Influence of IN-1 antibody and acidic FGF-fibrin glue on the response of injured corticospinal tract axons to human Schwann cell grafts. J. Neurosci. Res..

[77] Kuo H.-S., Tsai M.-J., Huang M.-C., Chiu C.-W., Tsai C.-Y., Lee M.-J., Huang W.-C., Lin Y.-L., Kuo W.-C., Cheng H. (2011). Acid fibroblast growth factor and peripheral nerve grafts regulate Th2 cytokine expression, macrophage activation, polyamine synthesis, and neurotrophin expression in transected rat spinal cords. J. Neurosci..

[78] Zhang R., Tu Y., Zhao M., Chen C., Liang H., Wang J., Zhang S., Li X. (2015). Preparation of Bionic Collagen-Heparin Sulfate Spinal Cord Scaffold with Three-dimentional print technology. Zhongguo Xiu Fu Chong Jian Wai Ke Za Zhi.

[79] Chen B., Bohnert D., Borgens R.B., Cho Y. (2013). Pushing the science forward: Chitosan nanoparticles and functional repair of CNS tissue after spinal cord injury. J. Biol. Eng..

[80] Nomura H., Baladie B., Katayama Y., Morshead C.M., Shoichet M.S., Tator C.H. (2008). Delayed implantation of intramedullary chitosan channels containing nerve grafts promotes extensive axonal regeneration after spinal cord injury. Neurosurgery.

[81] Seth N., Simmons H.A., Masood F., Graham W.A., Rosene D.L., Westmoreland S.V., Cummings S.M., Gwardjan B., Sejdic E., Hoggatt A.F. (2018). Model of Traumatic Spinal Cord Injury for Evaluating Pharmacologic Treatments in Cynomolgus Macaques (*Macaca fasicularis*). Comp. Med..

